# Giant aneurysm of the circumflex artery

**DOI:** 10.1002/ccr3.6521

**Published:** 2022-11-16

**Authors:** Dimos Karangelis, Christos Alexiou, Konstantinos C. Christodoulou, Zisis Gerontitis, Dimitrios Mikroulis

**Affiliations:** ^1^ Department of Cardiac Surgery Democritus University of Thrace, University Hospital of Alexandroupolis Alexandroupolis Greece; ^2^ Department of Cardiac Surgery Mediterraneo Hospital Glyfada Greece

**Keywords:** cardiac tamponade, coronary aneurysm, giant aneurysm, urgent surgery

## Abstract

In this paper, we describe a rare case of a giant aneurysm of the circumflex artery that we managed. A 59‐year‐old female patient presented in cardiogenic shock after partial aneurysm rupture. Giant aneurysms of the circumflex artery are extremely rare entities. The optimal surgical management dictates meticulous preoperative planning and the operation should be carried out on an elective basis.

## Abstract 2

Surgery for giant coronary aneurysms is a challenge. It should be carried out electively to allow detailed imaging and meticulous preoperative planning. Acute cardiac tamponade caused by spontaneous aneurysm rupture carries significant mortality.

## 1 INTRODUCTION

Coronary artery aneurysm (CAA) is defined as coronary dilatation, which exceeds the diameter of the normal adjacent artery segments or the diameter of the patient's largest coronary artery by 1.5 times.^1^ The “giant” CAA definition is still controversial; however, according to the Committee of the American Heart Association, giant aneurysms are defined as those >8 mm.^1^ The incidence of coronary artery aneurysms ranges between 1.5% and 5%, while the right coronary artery (RCA) is affected more frequently than the left anterior descending (LAD) and left circumflex artery (LCx).^2^ Herein we describe the surgical management of a 59‐year‐old female patient who presented urgently with a giant coronary aneurysm of the circumflex artery.

## 2 CASE REPORT

**FIGURE 1:**
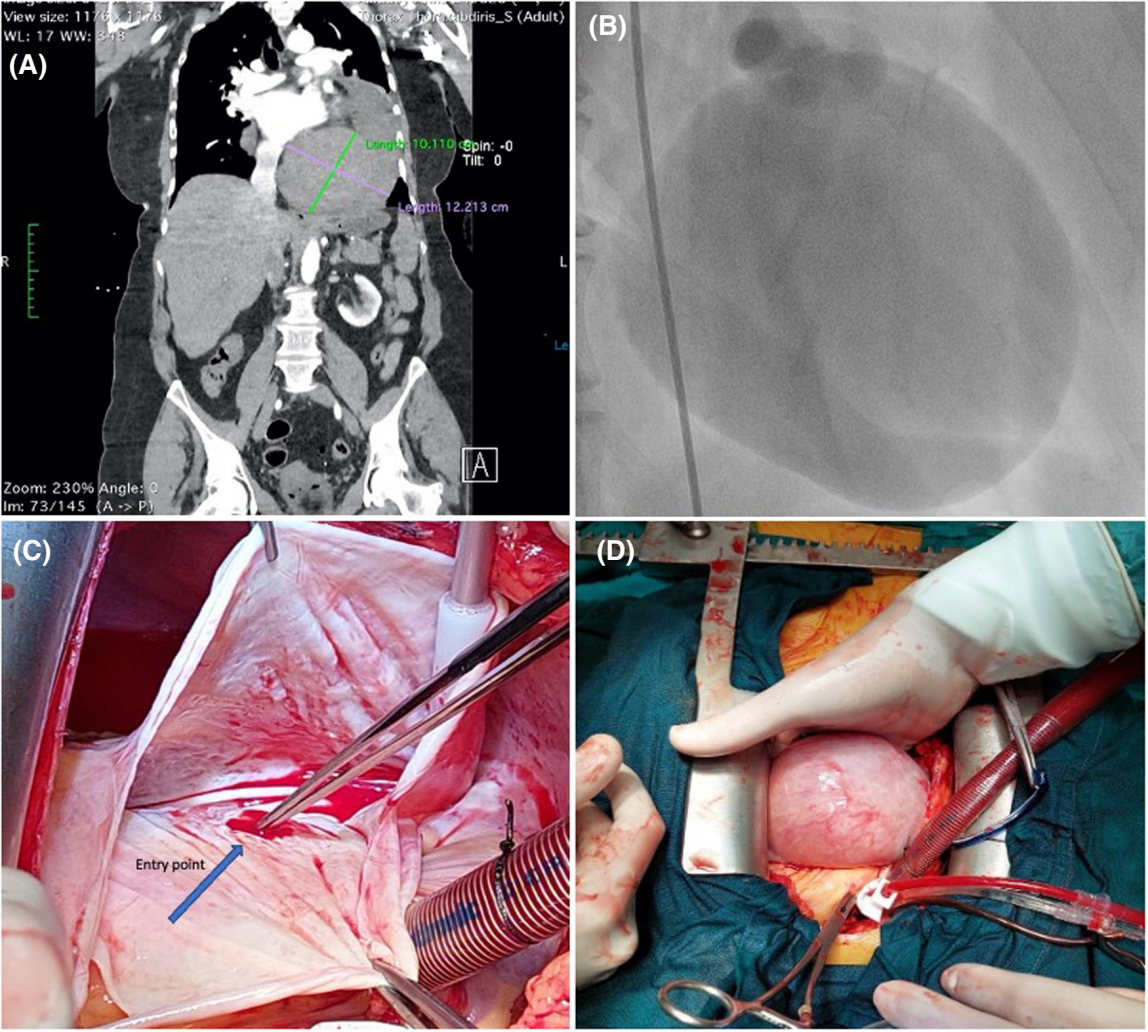
(A) Preoperative CT. The aneurysm measured 10 × 12 cm. (B) Coronary angiography. (C) The aneurysmal sac was opened, and the inflow (entry) point is visible. (D) Intraoperative image of the aneurysm (from patient's head).

The patient was urgently transferred to our department for management of a giant aneurysm of the LCx measuring 10 by 12 cm (Figure 1A,B). The patient was on another hospital's surgical list waiting to be operated. The operation had delayed due to COVID‐related issues, and the patient was experiencing daily angina‐like symptoms. Upon arrival, the patient was in a critical condition due to cardiac tamponade, requiring high doses of inotropic support. She underwent urgent cardiac surgery. The operation was conducted under cardiopulmonary bypass, which was established via femoral aortic and venous access. The venous cannula was later transitioned to a two‐stage right atrial cannula due to poor drainage. After median sternotomy, 500 ml of blood and clots were removed from the pericardial sac. The aneurysm had spontaneously ruptured and involved the left circumflex coronary artery, occupying most of the inferior surface of the left ventricular wall, and its size was displacing the heart superiorly and anteriorly, causing compression to the adjacent arteries. The patient's preoperative echocardiogram, besides the tamponade, was evident of a reduced ejection fraction. The aneurysmal sac was opened, and the inflow and outflow points were suture ligated (Figure 1C). A vein graft was anastomosed end‐to‐side to the coronary artery distally, while the aneurysmal cavity was obliterated by multiple sutures. The patient was weaned from cardiopulmonary bypass requiring high doses of inotropic support and intra‐aortic balloon pump assistance. She succumbed several hours later in the intensive care unit.

## 3 DISCUSSION

Giant CAAs, often defined as a CAAs over 2 cm in diameter, are extremely rare entities, and their incidence is reported to be around 0.02%.^3^ Despite the fact that etiology of CAAs is heterogeneous, the underlying mechanism remains vessel wall weakening and subsequent dilatation.^3^ Multiple factors have been incriminated for CAAs formation, such as percutaneous coronary intervention (iatrogenic), coronary artery disease, Kawasaki disease and Takayasu arteritis (vasculitis), Marfan disease (connective tissue disorder), and infections (mycotic aneurysms). Although most CAAs are often recognized during coronary angiography, additional diagnostic modalities have been employed for the imaging of CAAs such as echocardiography, cardiac computed tomography (CT) scan, and cardiac magnetic resonance imaging (CMR).^3^

Patients suffering from coronary aneurysms are often asymptomatic. Nevertheless, sometimes depending on the size of the aneurysm, they may present with symptoms of angina.^4^ Coronary aneurysms may be complicated with rupture, thrombosis, embolism, and fistula to the cardiac chambers.^4^ Most giant coronary aneurysms reported in the medical literature have involved the RCA adjacent to the right atrium.^5^ Surgical intervention for all coronary aneurysms is considered the mainstay of treatment, in view of the high risk for thrombosis and rupture. Resection of coronary artery aneurysm with coronary artery bypass (CABG) is the most frequently chosen treatment modality for giant aneurysms.

To the best of our knowledge, this is the one of the largest giant aneurysms ever reported in literature, involving the circumflex artery (Figure 1D).

## 4 CONCLUSION

Surgical management for giant aneurysms is a challenge; therefore, it should be carried out electively to allow detailed imaging and meticulous preoperative planning. Acute cardiac tamponade caused by spontaneous artery rupture carries significant mortality.

## AUTHOR CONTRIBUTIONS

Dimos Karangelis wrote the paper. Christos Alexiou assisted in writing. Konstantinos C. Christodoulou data acquisition and literature review. Zisis Gerontitis assisted in literature review and drafting. Dimitrios Mikroulis approved the final draft.

## FUNDING INFORMATION

None.

## CONFLICT OF INTEREST

None.

## CONSENT

Written informed consent was obtained from the patient to publish this report in accordance with the journal's patient consent policy.

## Data Availability

None.
